# Purkinje Cell Pathology and Loss in Multiple Sclerosis Cerebellum

**DOI:** 10.1111/bpa.12230

**Published:** 2014-12-31

**Authors:** Juliana Redondo, Kevin Kemp, Kelly Hares, Claire Rice, Neil Scolding, Alastair Wilkins

**Affiliations:** ^1^ Multiple Sclerosis and Stem Cell Group School of Clinical Sciences University of Bristol Bristol UK

**Keywords:** cerebellum, multiple sclerosis, neurofilament, Purkinje cell, spheroid

## Abstract

Cerebellar ataxia commonly occurs in multiple sclerosis, particularly in chronic progressive disease. Previous reports have highlighted both white matter and grey matter pathological changes within the cerebellum; and demyelination and inflammatory cell infiltrates appear commonly. As Purkinje cell axons are the sole output of the cerebellar cortex, understanding pathologic processes within these cells is crucial to develop strategies to prevent their loss and thus reduce ataxia. We studied pathologic changes occurring within Purkinje cells of the cerebellum. Using immunohistochemic techniques, we found changes in neurofilament phosphorylation states within Purkinje cells, including loss of dephosphorylated neurofilament and increased phosphorylated and hyperphosphorylated neurofilament. We also found Purkinje axonal spheroids and Purkinje cell loss, both of which occurred predominantly within areas of leucocortical demyelination within the cerebellar cortex. These changes have important implications for the study of cerebellar involvement in multiple sclerosis and may help design therapies to reduce the burden of ataxia in the condition.

## Introduction

The cerebellum and its efferent and afferent pathways are commonly affected in multiple sclerosis (MS). In patients with established MS, ataxia occurs in about 80% with symptoms and is particularly prevalent in those with progressive disease 24. Both cerebellar tremor and dysarthria may be found commonly in advanced disease.

Cerebellar white matter lesions are commonly found and are often apparent in magnetic resonance imaging (MRI) scans of patients with MS. Recent observations concerning grey matter demyelination in cerebral cortex have led to studies evaluating grey matter disease in the cerebellum 14, 17. Indeed, the cerebellar cortex appears a major site for demyelination with one study reporting 38.7% of the cerebellar cortex being affected in a cohort of primary progressive multiple sclerosis (PPMS) and secondary progressive multiple sclerosis (SPMS) patients 9. The same study also showed neuronal pathology with some reductions in Purkinje cell density in lesions (compared with control). No significant reductions in Purkinje cell densities were seen in non‐lesional cerebellar grey matter. Other changes in Purkinje cell phenotype have been documented in MS, notably changes in ion channel expression and receptor profiles. The Na_v_1.8 sensory neuron‐specific sodium channel is normally expressed at very low levels in Purkinje cells, but its expression is markedly up‐regulated in MS together with annexin light chain (p11), which facilitates the functional expression of this sodium channel 1, 2.

Purkinje cells represent the sole output neuron of the cerebellar cortex and thus changes in their function have significant impact on the function of the cerebellum as a whole. The aims of this study were to further characterize Purkinje cell pathology in MS cerebellum particularly with respect to neurofilament phosphorylation states, in light of descriptions of neurofilament abnormalities within white and grey matter of the cerebral hemispheres in MS 5, 26. We show increases in neurofilament hyperphosphorylation, loss of dephosphorylated neurofilaments, axonal spheroids and Purkinje cell loss, all of which are linked to lesion formation in the cerebellar cortex.

## Materials and Methods

### Cerebellar tissue

Post‐mortem cerebellar samples from five control cases and six patients with MS were obtained from the UK Multiple Sclerosis Tissue Bank at the Imperial College London, UK as previously described 6. The clinical background (age, sex, duration of disease, classification of MS, cause of death) of control and MS cohort are present in Table 1. All patients had been clinically diagnosed as having MS and this diagnosis had been confirmed during neuropathologic autopsy examination. Control cerebellum samples were derived from patients who had died from causes not linked to neurologic diseases. Brains were removed, fixed in formalin and embedded in paraffin. Sections of 10 μm in thickness were cut from cerebellar tissue and mounted onto glass slides.

**Table 1:** Clinical background of control and multiple sclerosis cohort

Patient	Age (years)	Sex (M/F)	Cerebellar lesion	Duration of disease (years)	Classification of MS	Cause of death
Control	82	M	Negative	0	n/a	Not known
Control	88	M	Negative	0	n/a	Prostate cancer, bone metastases
Control	68	M	Negative	0	n/a	Heart failure, fibrosing alveolitis, coronary artery artheroma
Control	84	M	Negative	0	n/a	Bladder cancer, pneumonia
Control	82	M	Negative	0	n/a	Myelodysplastic syndrome, rheumatoid arthritis
Mean	81			0		
MS 1	78	F	Chronic inactive	42	Secondary progressive	Metastatic carcinoma of bronchus
MS 2	64	F	Chronic active	36	Secondary progressive	Gastrointestinal bleed/obstruction, aspiration pneumonia
MS 3	49	F	Chronic inactive	18	Secondary progressive	Chronic renal failure, heart disease, general decline
MS 4	49	F	Chronic inactive	23	Secondary progressive	Bronchopneumonia
MS 5	42	F	Active	6	Primary progressive	Bronchopneumonia
MS 6	44	M	Chronic active/active	10	Secondary progressive	Bronchopneumonia
Mean	54			23		

F = female; M = male; MS = multiple sclerosis; n/a = not applicable.

### DAB staining on paraffin sections

DAB (3,3'‐Diaminobenzidine) staining for myelin basic protein (MBP) (1:3200, Serotec, Oxford, UK) and the macrophage/microglial markers (DP, DQ and DR subregions of MHC class II) (1:800, Dako, Cambridgeshire, UK) were performed in cerebellar sections of MS and control tissue, according to previously published reports from our laboratory 21. MBP staining was used to identify non‐demyelinated areas (MBP positive) from areas with cerebellar demyelination (MBP negative); while HLA‐DP, ‐DQ and ‐DR were used to detect inflammatory cell infiltrates. Images were acquired using an Olympus IX70 microscope coupled with (Media Cybernetics, Rockville, MD, USA).

### Immunofluorescence staining on paraffin sections

Paraffin‐embedded sections were double‐stained as previously described 6. Primary antibodies used were rabbit anti‐Calbindin‐D28K (1:500, Sigma‐Aldrich, Dorset, UK) for Purkinje cell identification; mouse monoclonal antibody to phosphorylated neurofilaments SMI‐31 (1:500, Covance, New Jersey, USA); mouse monoclonal anti‐hyperphosphorylated neurofilament SMI‐34 (1:500, Covance); and mouse monoclonal SMI‐32 (1:500, Covance) that reacts with non‐phosphorylated epitopes of neurofilaments. Species‐specific Alexa Fluor^®^ 488 and 555 conjugated secondary antibodies (1:500, Life Technologies, Paisley, UK) were used to visualize primary antibody staining. DAPI (4',6‐diamidino‐2‐phenylindole) Vectasheild™ (H‐1200, Vector Laboratories, Peterborough, UK) was used for nuclear identification. Sections were visualized using a Leica (DMI 6000) fluorescence light microscope with a Leica LAS‐AF software and also a Leica TCS SP5‐AOBS confocal laser scanning microscope attached to a Leica DMI 6000 inverted epifluorescence microscope to obtain three‐dimensional images.

### Quantification of demyelination and Purkinje cells in MS and control cerebellum

**Figure 1:**
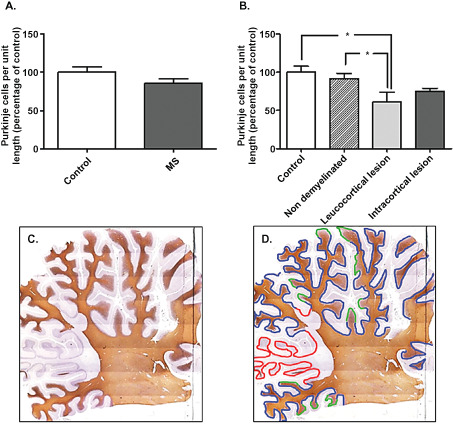
Purkinje cells quantification in control and MS cerebellum. The percentage of Purkinje cells per standardized unit length was similar between control and MS cases (**A**), but significantly reduced when comparing either control cases or non‐demyelinated areas with leucocortical lesion of MS. (**B**) Based on MBP staining, **C** sections were divided into non‐demyelinated areas (**D** blue), areas with leucocortical lesions (**D** red) and areas with intracortical lesions (**D** green) to perform counting. Results are expressed as the mean ± standard error, *P < 0.05.

The total number of Purkinje cells was counted in MS and control section, scanning the entire length of the Purkinje cell layer. Purkinje cells were identified based on Calbindin positivity and their specific location. To account for the difference in section sizes and plane of cutting, the length of the Purkinje cell layer was traced and measured in each section using Image J software to obtain the percentage of Purkinje cells per standardized unit length. For these sections, the length of Purkinje cell layer was measured and the number of Purkinje cells in each area counted. Additionally, MS sections were subdivided into non‐demyelinated (Figure 1D blue), leucocortical lesion (Figure 1D red) and intracortical lesion (Figure 1D green) based on the MBP staining (Figure 1C) and dependent on whether the region of demyelination was adjacent to the Purkinje cell layer. For each MS section, the total length of the Purkinje cell layer was measured as earlier. The length of demyelinated regions (leucocortical and intracortical) were also measured to obtain the percentage of demyelinated regions in comparison with the total section.

### SMI and spheroids quantification in MS and control cerebellum

**Figure 2:**
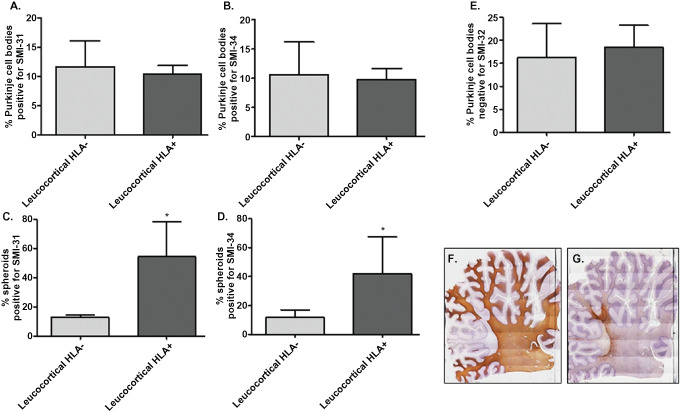
Relationship between SMI changes/spheroid formation and inflammation in MS. Regions with leucocortical lesions of MS sections **F** were divided based on the presence/absence of inflammatory cells infiltrates using the markers HLA‐ DP, ‐DQ and ‐DR. (**G**) No significant difference were observed in the frequency of Purkinje cells bodies stained for SMI‐31 (**A**) SMI‐34 (**B**) and SMI‐32 (**E**) between demyelinated areas positive or negative for HLA. The frequency of spheroids positive for SMI‐31 (**C**) and SMI‐34 (**D**) were increased in leucocortical lesions positive for HLA compared with lesions without inflammation. Results are expressed as the mean ± standard error, *P < 0.05.

The total number of spheroids, Purkinje cells bodies positive for SMI‐31, Purkinje cells bodies positive for SMI‐34 and Purkinje cells bodies negative for SMI‐32 were counted in MS and control sections, scanning the entire Purkinje cell layer. As above, MS sections were subdivided into non‐demyelinated, leucocortical lesion, intracortical lesion regions and the frequency of spheroids and abnormal Purkinje cell body staining (SMI‐31/SMI‐34/SMI‐32) were counted and divided by the total number of Purkinje cells in the specific region. Furthermore, regions with leucocortical lesions were subdivided based on presence/absence of inflammatory cell infiltrates using the markers HLA‐DP, ‐DQ and ‐DR (Figure 2F,G).

### Statistic analysis

Statistic analysis was performed using (GraphPad Software, La Jolla, CA, USA). Data were analyzed using one‐way analysis of variance (ANOVA) with post hoc Bonferroni's test between groups where appropriate and unpaired *t*‐test or Mann–Whitney test on parametric or non‐parametric data, respectively. Values are expressed as the mean ± standard error and differences at *P* < 0.05 represent statistic significance.

## Results

### Distribution of SMI‐31, SMI‐34 and SMI‐32 in cerebellar cortex of MS and control patients

To investigate whether abnormal neurofilament phosphorylation was present in Purkinje cell bodies of MS patients, cerebellar sections from control and MS cases were double immunostained. Antibodies used were the Purkinje cell marker Calbindin with SMI‐31 (anti‐phosphorylated neurofilament), SMI‐34 (anti‐hyperphosphorylated neurofilament) or SMI‐32 (anti‐non‐phosphorylated neurofilament).

**Figure 3:**
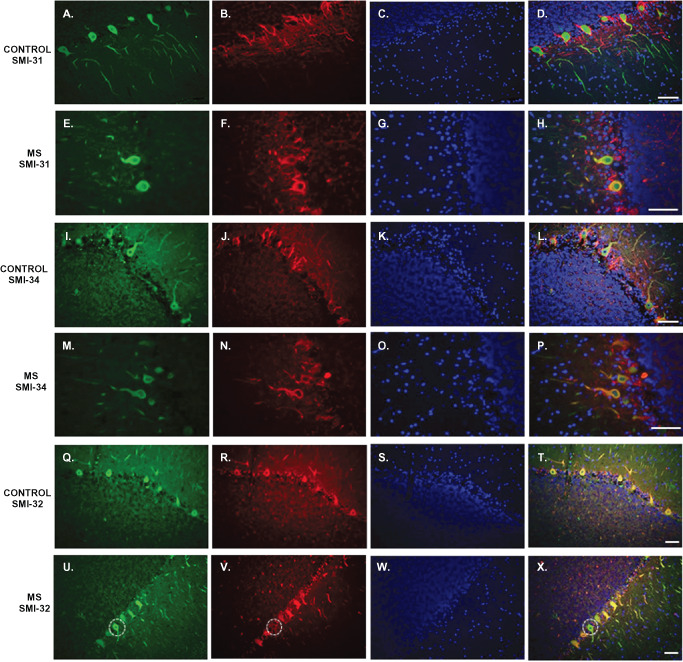
Abnormal SMI‐31, SMI‐34 and SMI‐32 staining of Purkinje cells in MS cerebellum. Cerebellar sections derived from control cases (**D,L,T**) and patients with multiple sclerosis (**H,P,X**) were immunolabelled with Calbindin‐D28K (green), SMI‐31/SMI‐34/SMI‐32 (red) and the nuclear marker DAPI (blue). In control sections, Purkinje cells bodies were negative for SMI‐31 (**B**) and SMI‐34 (**J**); while in MS sections a number of Purkinje cells bodies were positive for both these markers (**F,N**). SMI‐32 stained almost all Purkinje cells bodies in control sections (**R**), while in MS cases there was loss of expression in some Purkinje cells bodies (**V**). Scale bar: 100 μm.

In control sections, Purkinje cell bodies were negative for SMI‐31 (Figure 3B) and SMI‐34 (Figure 3J). Staining for both SMI‐31 and SMI‐34 was seen in basket cell axons around Purkinje cell bodies and in parallel fibers (axons of granule cells) in the molecular layer (Figure 3D,L). In sections from MS patients, a number of Purkinje cell bodies were positive for both SMI‐31 (Figure 3F) and SMI‐34 (Figure 3N). An overall decrease of SMI‐31 and SMI‐34 labeling of surrounding cell processes was also observed in MS sections (Figure 3H,P).

Immunostaining using SMI‐32 (directed against non‐phosphorylated neurofilaments) in control cerebellum revealed that almost all Purkinje cells bodies were positive for this marker (Figure 3R); while in MS sections, there was a loss of expression in a number of Purkinje cell bodies (Figure 3V).

### Spheroid formation and pathologic abnormalities in MS cerebellum

**Figure 4:**
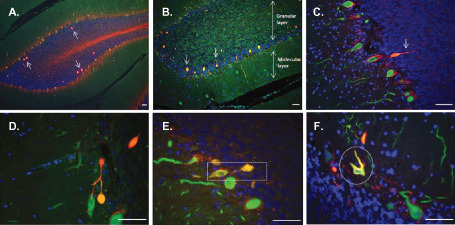
Spheroid formation and pathologic abnormalities in MS cerebellum. Immunostaining for Calbindin‐D28K (green), SMI‐31/SMI‐34 (red) and the nuclear marker DAPI (blue) revealed pathologic changes in cerebellar sections from MS cases. In the granular layer spheroids (see arrows) positive for SMI‐31 (**A**) and SMI‐34 (**B**) were observed. Purkinje cell axons in continuity with spheroids (arrow **C,E**) and axonal collaterals (**D**) were also seen. Purkinje cells body positive for SMI‐34 often displayed an abnormal morphology with a less flask‐like shape (**E,F**). Scale bar: 100 μm.

Double immunostaining for Calbindin and SMI‐31/SMI‐34 revealed other pathologic changes in sections from MS cases, besides the abnormal Purkinje cell body staining. In the granular layer of MS cerebellum (immediately adjacent to the Purkinje cell layer) spheroids were observed (Figure 4A,B) These spheroids were ovoidal structures, negative for SMI‐32, but strongly immunoreactive for Calbindin and both SMI‐31 (A) and SMI‐34 (B), suggesting that they consist of accumulated phosphorylated and hyperphosphorylated neurofilament. In some cases, a segment of axon was visible in continuity with the spheroid (Figure 4C) and some axonal collaterals were seen (Figure 4D) confirming that these structures represent axonal spheroids. Furthermore, Purkinje cells bodies positive for SMI‐34 often displayed an abnormal morphology, with a less flask‐like shape (Figure 4E,F).

**Figure 5:**
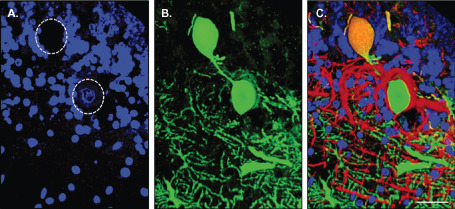
Purkinje cell axonal spheroid in MS. A single Purkinje cell from MS cerebellum stained for Calbindin‐D28K (green), SMI‐34 (red) and the nuclear marker DAPI (blue) was visualized with confocal microscopy. The three‐dimesional image confirmed that the spheroid arose from the proximal portion of the Purkinje cell axon (**B**). The spheroid structure had no nucleus (**A**) and was positive for SMI‐34 (**C**). Scale bar: 50 μm.

Spheroids appeared to arise from the proximal portion of the Purkinje cell axon (Figure 4E). MS sections were therefore viewed using a confocal microscope. From the three‐dimensional image, it was possible to visualize the Purkinje cell body, its axon and the axonal spheroid all in continuity (Figure 5B). The spheroid structure indeed had no nucleus (Figure 5A) and was positive for SMI‐34 (Figure 5C).

### Quantification of demyelination in MS cases

Areas of cerebellar demyelination were present in all MS cases and not seen in controls. Two main types of demyelination were seen: demyelination of cerbellar cortex in continuity with demyelination in the white matter (leucocortical); and demyelination restricted to cerebellar cortex and not expending to white matter (intracortical). MS sections were subdivided into non‐demyelinated regions (Figure 1D blue), leucocortical lesions (Figure 1D red) and intracortical lesions (Figure 1D green), based on the MBP staining (Figure 1C). The entire length of the Purkinje cell layer and the length of demyelinated regions of each MS case were measured. 29.4% [±11.5 standard error of the mean SEM] of cerebellar cortex showed demyelination, representing 13.7% (±8.5 SEM) with leucortical lesions and 15.7% (±7.2 SEM) with intracortical lesions.

### Purkinje cell number in MS and control cerebellum

In order to determine whether there was a decrease in Purkinje cell number in MS cases compared with control, the entire length of Purkinje cell layer was measured for each section and Purkinje cells counted to obtain the percentage of Purkinje cells per standardized unit length. There was no significant difference in Purkinje cell number in MS cerebellum compared with control sections (Figure 1A). Interestingly, the number of Purkinje cells were similar between control and non‐demyelinated areas of MS cases, but significantly reduced when comparing either control cases or non‐demyelinated regions with leucocortical lesion regions of MS (Figure 1B; *P < 0.05). The Purkinje cell number within intracortical lesions was slightly reduced compared with non‐demyelinated areas of MS and control cases, but this reduction was not significant (Figure 1B).

### Quantification of spheroids and SMI staining in MS and control cerebellum

**Figure 6:**
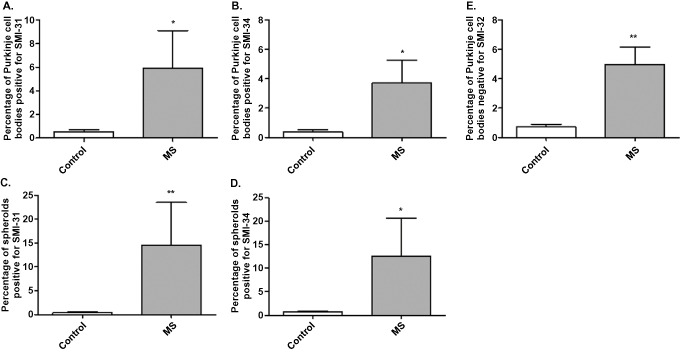
Quantification of SMI staining and spheroids in MS and control cerebellum. A significant increase in the number of Purkinje cell bodies positive for SMI‐31 (**A**) and SMI‐34 (**B**) and spheroids (**C,D**) were observed in MS patients compared with control cases. SMI‐32 cell body staining was decreased in MS cases, with a significantly higher number of Purkinje cell bodies negative for SMI‐32 in MS compared with controls (**E**). Results are expressed as the mean ± standard error, *P < 0.05; **P < 0.01.

**Figure 7:**
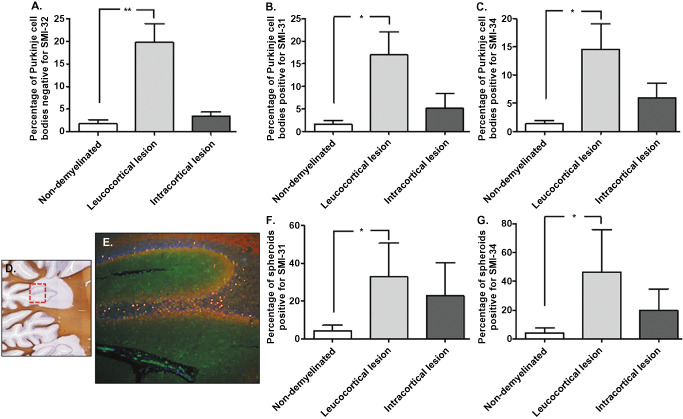
Relationship between SMI changes/spheroid formation and demyelination in MS. A significant increase in Purkinje cell bodies positive for SMI‐31 (**A**) and SMI‐34 (**B**) and negative for SMI‐32 (**C**) were observed in leucocortical lesions compared with non‐demyelinated areas of MS cases. Low magnification of immunostaining for Calbindin‐D28K (green) and SMI‐31 (red) (**E**) showed high numbers of spheroids in a demyelinated area (**D**). The number of spheroids positive for SMI‐31 (**F**) and SMI‐34 (**G**) were higher in leucocortical lesions compared with non‐demyelinated areas of MS. Purkinje cell bodies staining for SMI31/34 and the frequency of spheroids in areas with intracortical lesions were increased comparing with non‐demyelinated areas, although not significant. Results are expressed as the mean ± standard error, *P < 0.05; **P < 0.01.

For each section, the number of Purkinje cells bodies and spheroids positive for SMI‐31 and SMI‐34 were counted. There was a significant increase in the number of spheroids (Figure 6C,D; *P < 0.05, **P < 0.01) and Purkinje cells bodies positive for SMI‐31 (Figure 6A; *P < 0.05) and SMI‐34 (Figure 6B; *P < 0.05) in MS patients compared with control cases. SMI‐32 cell body staining was decreased in MS cases, with a significantly higher number of Purkinje cells bodies negative for SMI‐32 in MS compared with controls (Figure 6E; **P < 0.01). No spheroids were positive for SMI‐32. MS sections were subdivided into non‐demyelinated regions, leucocortical lesions and intracortical lesions. Low magnification of the double‐immunostaining for calbindin and SMI‐31 (Figure 7E) showed high numbers of spheroids in demyelinated areas (Figure 7D). Indeed, the frequency of spheroids (Figure 7F,G; *P < 0.05) and Purkinje cell bodies positive for SMI‐31 (Figure 7A; *P < 0.05) and SMI‐34 (Figure 7B; *P < 0.05) were higher in the leucocortical lesions compared with non‐demyelinated areas of MS cases. In accordance, in areas with leucocortical lesions, a significant number of Purkinje cells were negative for SMI‐32 cell body staining compared with non‐demyelinated areas (Figure 7C; **P < 0.01). In intracortical lesions of MS cases, Purkinje cell bodies staining for SMI31/34 and the frequency of spheroids were increased compared with non‐demyelinated areas although not significant (Figure 7B,C,F,G).

### Correlation between number of spheroids and inflammation in demyelinated areas of MS cerebellum

To investigate whether inflammation could have a role in spheroid formation or in the abnormal Purkinje cell body staining, MS sections were further studied. Leucocortical lesions negative for MBP (Figure 2F) were divided based on presence/absence of a marked inflammatory cell infiltrate, according to the HLA‐DP, ‐DQ and ‐DR staining (Figure 2G). Interestingly, spheroids positive for SMI‐31 (Figure 2C; *P < 0.05) and SMI‐34 (Figure 2D; *P < 0.05) were present with a high frequency in leucocortical lesions positive for HLA compared with lesions without inflammation, suggesting that spheroid formation may be driven by inflammation. No significant differences were present in the frequency of Purkinje cell body staining for SMI‐31 (Figure 2A), SMI‐34 (Figure 2B) and SMI‐32 (Figure 2E) between demyelinated areas positive or negative for HLA.

## Discussion

Symptoms of cerebellar dysfunction—ataxia, dysarthria and tremor—are common manifestations of MS. In patients with established progressive disease, cerebellar symptoms are difficult to treat and generally worsen as time progresses. Understanding key pathologic changes in the cerebellum may help design therapies for progressive ataxia in the disease. In this report, we show loss of Purkinje cells within demyelinated areas of cerebellar cortex. In addition, we show a number of pathologic changes including loss of dephosphorylated neurofilament labeling in Purkinje cells, increases in phosphorylated and hyperphosphorylated neurofilament staining in Purkinje cells and Purkinje axonal spheroids.

The cerebellar cortex appears a major site for demyelination in MS with one study reporting 38.7% of the cerebellar cortex being affected in a cohort of PPMS and SPMS patients 9. Interestingly, in this study, many grey matter lesions appeared independent of white matter lesions, with some tissue blocks showing very extensive grey matter demyelination in the near absence of underlying white matter disease. The same study also showed reductions in Purkinje cell density in lesional grey matter (compared with control), but no significant reductions in Purkinje cell densities were seen in non‐lesional cerebellar grey matter. The current study has confirmed this finding.

In addition, we have shown pathologic changes within Purkinje cells that appear most marked within demyelinated areas. Neurofilament changes in MS have been documented in the cerebral cortex and white matter tracts. Neuronal and axonal cytoskeletons are composed of variably phosphorylated neurofilaments of low, medium and high molecular weight (MW) that perform many diverse cellular functions, but are predominantly involved in providing structural stability 10, 21. Neurofilament (NF) subunits are the most extensively phosphorylated proteins in neurons and this phosphorylation is highly regulated, with an intense phosphorylation in axons and little or no phosphorylation in cell bodies and dendrites 16. Antibodies directed against different neurofilament phosphoisoforms have long been used to study changes in neurofilament phosphorylation as a marker of axonal pathology in several neurologic diseases. In MS, reductions in neurofilament phosphorylation within white matter axons are found, most markedly within lesions 26. In addition, neurons containing hyperphosphorylated (SMI‐34 staining) are found in MS cerebral cortex 5. Furthermore, the correlation of clinical disability with elevated neurofilament phosphoisoforms in cerebrospinal fluid (CSF) of MS patients highlights its importance as a biomarker for axonal and neuronal injury 18. Abnormal phosphorylation and accumulation of perikaryal neurofilaments has been found to occur also in Alzheimer's disease (AD) and amyotrophic lateral sclerosis (ALS), highlighting the critical role of neurofilament alteration in the settings of neurodegeneration 11. Accumulation of phosphorylated neurofilament in motor neurons are found in ALS 23; as well as a sevenfold elevation of phosphorylated NF heavy subunit (pNFH levels) in patient's CSF, supporting the potential use of pNFH as a new diagnostic biomarker 4. Neurofilament levels are altered also in AD 28 and neurofilaments found in neurofibrillary tangles are extensively phosphorylated 15. Furthermore, β‐amyloid plaques from patients in a preclinical stage of the disease are associated with abnormal accumulation of NF in the absence of tau abnormalities, suggesting that neurofilament changes may represent the earliest cytoskeleton alteration associated with dystrophic neurite formation 3, 27. In addition, loss of non‐phosphorylated neurofilament in neuronal cell bodies in the spinal cord from ALS 20 and temporal cortical areas in AD 25 has been documented.

The current study has revealed neurofilament phosphorylation changes in Purkinje cells, namely reduced numbers of cells staining for SMI‐32 (non‐phosphorylated NF) and increased numbers of cells staining for SMI‐31 and SMI‐34 (phosphorylated and hyper‐phosphorylated NF). Reduced staining for SMI‐32 in ventral spinal neurons has previously been documented in MS samples 20. In keeping with our previous study of MS cerebral cortex, we have found that neurofilament phosphorylation changes are most marked in Purkinje cells within demyelinated areas 5. Furthermore, we subdivided demyelinated areas into leucocortical lesions (cortical demyelination with demyelination in the adjacent white matter) and purely intracortical lesions. Abnormal phosphorylation was present in both lesions, but only significant for leucocortical lesions. It is not clear why Purkinje cells within leucocortical lesions are more susceptible to neurofilament changes. White matter plaques in continuity with demyelinated areas in the cortex may have a combined deleterious effect on Purkinje cells. Transected Purkinje cell axons in the white matter may lead to abnormal axonal transport to and from the cell body which may ultimately result in an abnormal accumulation of perikaryal neurofilament.

We have shown that Purkinje cell bodies positive for SMI‐34 displayed often an abnormal morphology, with a less flask‐like shape. Accumulation of hyperphosphorylated neurofilaments in Purkinje cells of the cerebellar cortex in MS may represent an important neurodegenerative mechanism. Although the precise relationship between hyperphosphorylated neurofilament accumulation and neuronal damage remains unknown, aberrant perikaryal accumulation may contribute to neuronal dysfunction and eventually to cell death.

Axonal spheroids (torpedoes) are found extensively within MS white matter lesions 26. Spheroids represent transected axons and are a common finding in a number of neuro‐inflammatory and neurodegenerative disorders. Spheroids arising from Purkinje cell axons have been noted in a number of ataxic conditions and in essential tremor 12, 13. Spheroids typically occur in the proximal portion of the Purkinje cell axon and may be a common response to cerebellar injury. The relationship between spheroid formation and Purkinje cell loss, however, appears complex, and it is not clear whether spheroid formation represent an early reversible process that precedes Purkinje cell loss 13. In this study, we have demonstrated Purkinje axonal spheroids, which stain for SMI31 and SMI34, with a clear increase in spheroid formation in demyelinated regions of the cerebellum, suggesting a relationship between demyelination, inflammation and axonal pathology.

Regions with leucocortical lesions were subdivided based on presence/absence of inflammatory cell infiltrates using the markers HLA‐DP, ‐DQ and –DR. This subdivision was not made for intracortical lesions as it was harder to define the inflammation within this lesion. This study has shown that spheroid formation in the cerebellar cortex appear most marked in leucocortical lesions with high levels of inflammatory cell infiltration. This suggests a possible link between axonal transection/spheroid formation and inflammation. In MS white matter, acute axonal injury (measured by amyloid precursor protein accumulation) is elevated in acute lesions and correlates to the degree of inflammation 8. In addition, levels of axonal transection are most marked in acute white matter lesions 26. Our data suggest injury to the proximal Purkinje cell axon with spheroid formation may also be linked to inflammatory cell activity. Our study, however, has shown that the presence of an inflammatory infiltrate was not associated with higher levels of abnormal Purkinje cell body neurofilament phosphorylation changes. There is likely to be a dynamic relationship between axonal spheroid formation, alteration in structural proteins (eg, phosphorylated neurofilaments) and Purkinje cell loss, which the current study, by nature of its neuropathologic material, cannot determine.

Other changes in Purkinje cell phenotype have been documented in MS, notably changes in ion channel expression and receptor profiles. The Na_v_1.8 sensory neuron‐specific sodium channel is normally expressed at very low levels in Purkinje cells, but its expression is markedly up‐regulated in MS 1. In addition, annexin light chain (p11), which facilitates the functional expression of this ion channel, is also up‐regulated in Purkinje cells 2. In experimental models, aberrant expression of Na_v_1.8 in Purkinje cells causes significant abnormalities of firing patterns of these cells 19, 22.

Optimal Purkinje cell function is vital to the overall function of the cerebellum, as Purkinje cell axons represent the sole output of the cerebellar cortex. Loss of Purkinje cells is a common finding in a range of neurologic conditions characterized by ataxia, and in all likelihood, is irreversible. Thus, designing therapies to protect Purkinje cells in MS and related conditions is vital to preserve cerebellar function. Recent reports of bi‐nucleate Purkinje cells and heterokaryon formation in experimental models and in human disease have suggested a potential intrinsic repair mechanism of Purkinje cells, whereby nuclear material is donated to an injured cell 6, 7. Understanding the precise pathologic changes in Purkinje cells will aid the search for reparative and regenerative therapeutic approaches.
